# Innovative and beneficial informal sweetpotato seed private enterprise in northern Uganda

**DOI:** 10.1007/s12571-017-0680-4

**Published:** 2017-05-19

**Authors:** Paul Rachkara, David Paul Phillips, Stephen Wamala Kalule, Richard William Gibson

**Affiliations:** 1 Department of Rural Development and Agribusiness, Gulu University, Gulu, Uganda; 2 Natural Resources Institute (NRI), University of Greenwich, London, UK

**Keywords:** Seed systems, Private enterprise, Smallholder agriculture, Vine marketing

## Abstract

Research conducted in the informal sweetpotato seed (vines) supply system in the Gulu region, northern Uganda (2013–2015) revealed a diverse set of actors using private enterprise in a range of selling and marketing channels. The different channels offer an efficient and effective marketing system, providing different services and conveniences for farmers at different prices. The actors include local vine multipliers, traders, dry season root farmers, transporters and town sellers. The local multipliers and dry season root farmers grow crops during the dry season in swampy areas and sell the vines in the following rainy season to the many farmers who lack access to such areas and therefore lack vines to plant. The presentation and discussion of this case study adds to an expanding argument in the literature for increased attention to support actors in informal food crop sectors who are providing sustainable production and marketing systems on a platform of beneficial and innovative private enterprise. Through their commercial operations, vine multipliers and other actors can effectively meet the demand of customers and at the right time and place. With suitable dissemination programmes installed, these actors could also offer access to new varieties otherwise unavailable to the majority of farmers.

## Introduction

Agriculture is a critical sector for most developing economies; commercial agriculture can drive economic development, improvements in incomes and livelihoods, and boost food security (IFAD 2003; DFID 2015). Seed systems ensuring farmers can access good quality planting material at the right time and of sufficient diversity are the foundation of agriculture, and support higher production, nutrition and resilient food system goals (McGuire and Sperling 2016). To date most of the seed that is commercially available for food crops in developing countries is that of cereals, especially hybrid maize. Also, much of the literature on seed systems is on those crops in which the seed is the botanical seed (Abay et al. 2011; Bellon and Brush 1994; Louette et al. 1997; Sperling and Berkowitz 1994; Tsehaye et al. 2006; Voss 1992) and emphasizes social relationships (Almekinders et al. 1994; McGuire 2007) rather than commerce. Most studies of seed systems of vegetatively propagated crops have involved cassava and potato. For cassava, they have mostly been anthropological (Boster 1986; Elias et al. 2001; Salick et al. 1997; Sambatti et al. 2001). For potato, a few have been anthropological (Brush 1992) but most have involved the formal system or its interrelationship with informal systems (Crissman et al. 1993; Etwire et al. 2013; International Potato Center 2011). This paper describes an informal sweetpotato seed system in Uganda, telling how the few farmers who have sweetpotato crops in lowlands during the dry season sold the vines onfarm, at local markets or through intermediaries including traders (middlemen) and sellers. It documents prices along supply chains, how much was traded and when, where and who purchased the vines. The paper focuses on enterprise and is the first well-documented and quantified description of an informal sweetpotato seed system in Africa.

In recent years there have been a number of publications discussing the contributions and potential for improving the distribution of good quality planting material to more end users through supporting the informal sector, and how stronger smallholder planting material enterprises can take new varieties from the formal sector to distribute through their networks (Neate and Guei 2010; McGuire and Sperling 2013; de Boef and Thijssen 2010; Louwaars and de Boef 2012, Samberg et al. 2013). This paper adds to the broad literature by presenting diverse systems of production, distribution and sale of planting material in the sweetpotato informal sector which, through appropriate support, can positively contribute to key objectives of food systems that include efficiency, welfare, safety nets, and food security (Timmer 2015). Previous sweetpotato seed projects, including those in northern Uganda (Namanda et al. 2007; Odongo et al. 2007), have largely ignored or been in ignorance of informal sweetpotato seed systems and instead have created new systems involving farmer field schools and/or decentralized vine multiplier groups (Stathers et al. 2005, 2013) whose produce is typically purchased by NGO or government contracts for free distribution to farmers. Few of these have thrived for long after projects have terminated (Gibson 2013). In contrast, our study, focusing on Gulu District in northern Uganda, sought to understand existing informal seed systems as a precursor to developing them in an evolutionary way. The presentation and discussion of this work demonstrates how seed entrepreneurs and farmers can operate in sustainable seed systems based on commerce compared to aid-based seed systems which depend on the whims of donors and seem likely to be unreliable in the long term.

## Context and definitions

Uganda is the fourth largest producer of sweetpotato in the world and second in East Africa with an estimated annual production of 2.6 m metric tonnes of fresh storage roots (henceforth simply called ‘roots’) from 550,000 ha (FAOSTAT 2013). Uganda’s formal seed industry has recently been transferred from public to private sector led (MAAIF 2014), in line with the enterprise spirit identified in this manuscript. The unregulated informal sector is also being considered for improvement, though a strategy for the introduction of Quality Declared Seed as a source of seed designed for farmers (Fajardo et al. 2010) has not yet been approved (MAAIF 2014).

Sweetpotato is widely grown for its roots which are used for household food and small-scale trading. It is propagated from vine cuttings and, in areas where a long dry season destroys vines of the main crop, farmers are willing to pay for vines as planting material (Anonymous 2009) and sales have been reported (Gibson et al. 2009; Namanda et al. 2011) though there is little detail of the type of farmer who is prepared to pay. Northern Uganda contributes about 16% of the national sweetpotato output, with Gulu District producing 62,000 MT, 21% of the region’s production (UBoS 2015). Gibson (2013) described three main kinds of sweetpotato seed systems operating in this region:

- A formal one dealing solely with released varieties, largely limited to research stations and with limited capacity to distribute them to farmers;
- A project-based (aid) one led by non-governmental organizations (NGOs), distributing free planting material of released varieties to smallholders;
- Informal ones characterized by lowland dry season production of vines for sale.

They also differ in their function:

- The formal system breeds and releases orange-fleshed (OFSP) and white-fleshed sweetpotato (WFSP) varieties;
- The aid seed system largely distributes OFSP varieties. Their roots are a source of vitamin A, especially valuable to nursing women and children (Kapinga et al. 2007; Low et al. 2007; Mwanga and Ssemakula 2011);
- Informal systems almost exclusively distribute landraces (Gibson 2013); most are white-fleshed (WF); some are high yielding and have been released by the formal system (Mwanga et al. 2001, 2009).

Formal seed systems in Uganda largely comprise research institutes and extension services sustained by government funding, sometimes using funds provided by international donors, for example, The World Bank and The International Fund for Agricultural Development funding the Ugandan National Agricultural Advisory Service (NAADS) (accessed 27 September 2016). Aid seed systems are sustained by projects; and informal systems by sales, barter and other forms of exchange. The informal seed systems comprise mainly local multipliers producing planting material for their own use and increasingly to sell to other farmers, sometimes through other actors.

**Fig 1:**
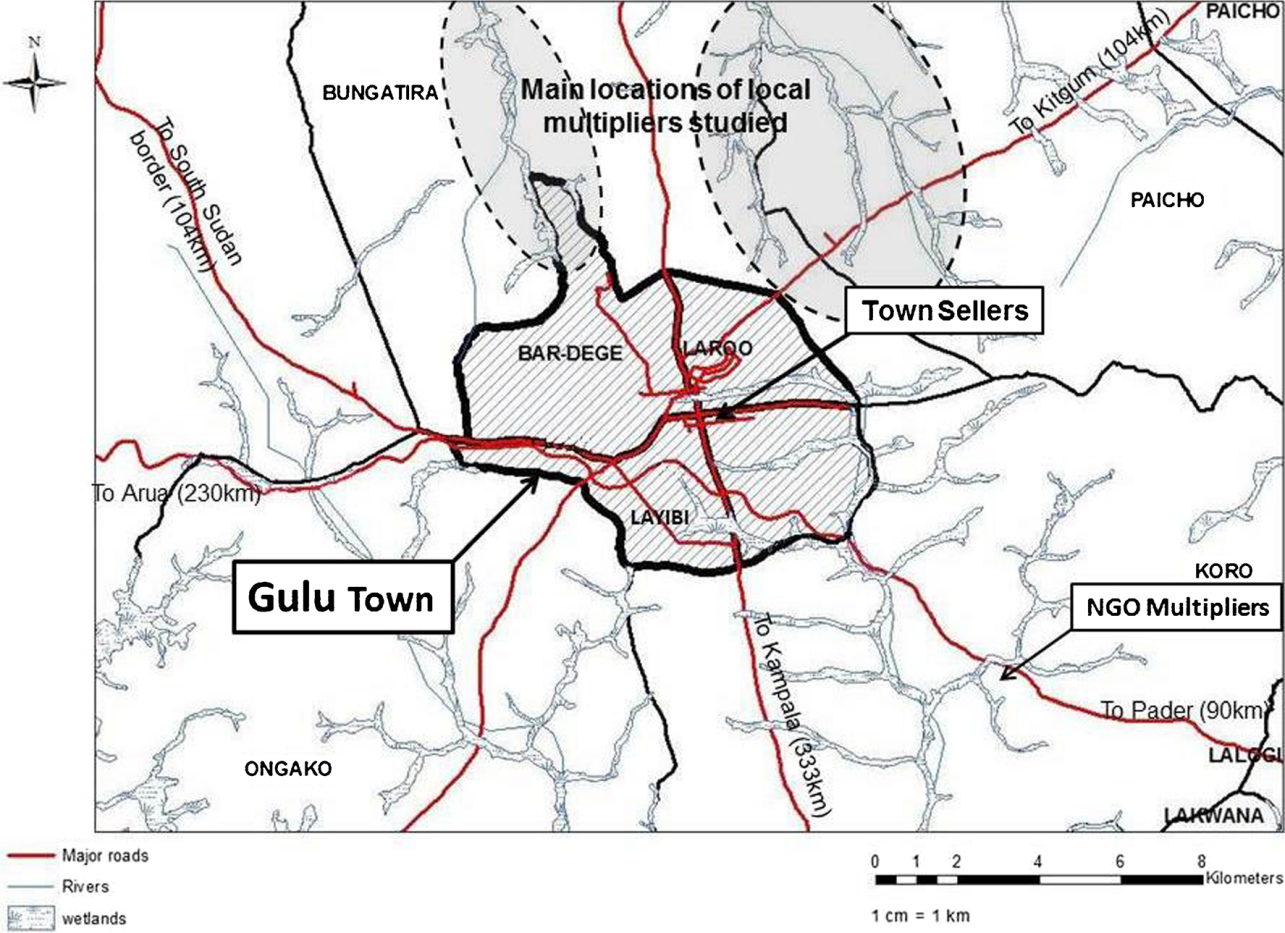
Map showing the locations of the local multipliers, NGO multipliers and town sellers in relation to Gulu Town and administrative districts, to the main swamps and river systems and to the transport network

At Gulu Town, roads leading east to Pader, north-east to Kitgum, north-west to Adjumani and west to Arua intersect the main road between Kampala and Juba, capitals and main cities of respectively Uganda and South Sudan (Fig. 1). There are frequent bus and minibus services to Kampala, Kitgum and South Sudan. Gulu has a high annual rainfall (1.5 m) and the main growing season is April–January, late planted crops maturing on residual soil moisture. At nearly 30 north, the long dry season lasts from December to mid-March. This is too long for sweetpotato crops not to desiccate and most farmers lack vines to plant at the start of the rainy season in April. Instead, the few farmers with land in swampy areas plant a crop there in December. The roots generated by this crop (April–June) are valuable as few other fresh foodstuffs are then available but the vines are now especially valuable and they are sold as planting material to the many farmers who lack access to swamps. This is the basis of the seed system.

## Method

**Fig 2:**
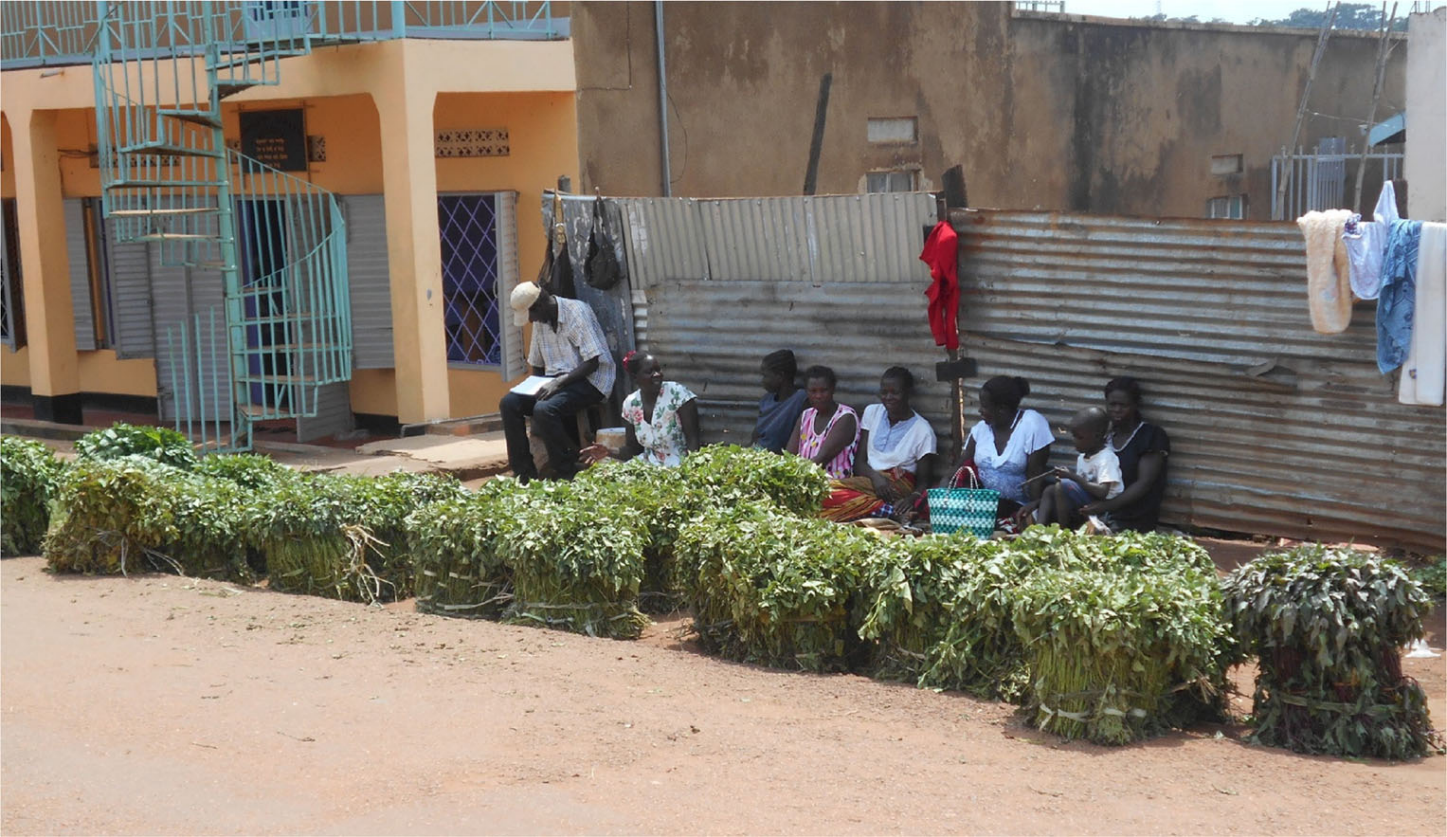
Vine sellers (together with our interviewer on extreme left) in Gulu Town selling large bundles of vines

**Fig 3:**
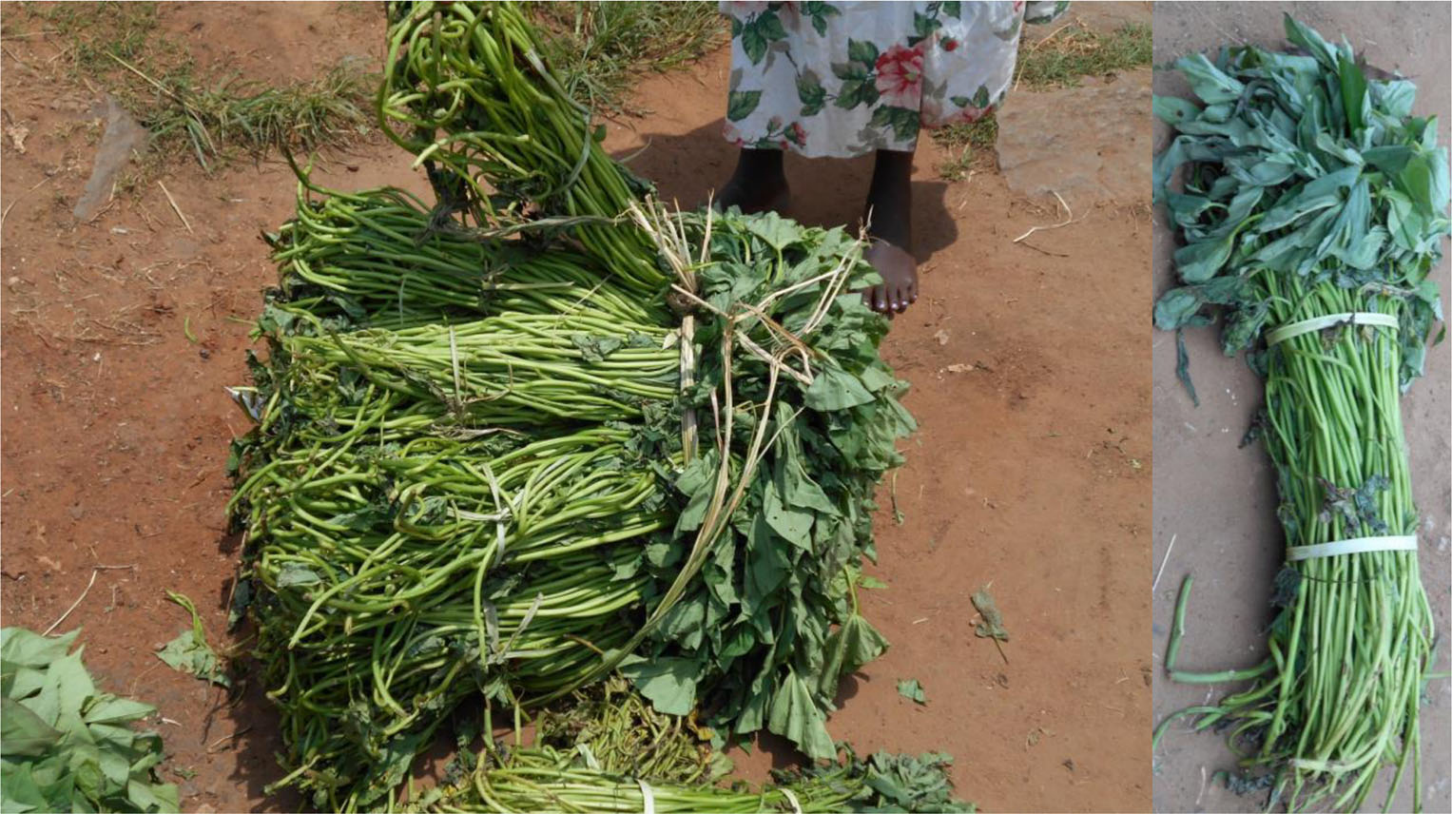
Photograph showing how each large bundle sold by town sellers is composed of 20 small bundles of 50 vine cuttings. The latter (inset on right) is the standard bundle used throughout the paper

During 2013, a few informal local multipliers and town sellers (Fig. 2) in and around Gulu Town were traced, initially through local knowledge and then, for 2014, from these original multipliers and sellers. Each year at the start of the selling season, these informal multipliers and sellers filled in a questionnaire on their gender, age, location and ownership of vine production areas, methods of production, varieties grown and access to a mobile phone. In 2013, 19 putative multipliers were identified but 5 had no sales by late April and were disregarded. In 2014, 56 multipliers were identified but only 27 were monitored, largely because others had either failed to plant in the dry season (it arrived unusually early) or they were unwilling to be monitored. All season-long sellers in Gulu Town were monitored, 7 in 2013 and 10 in 2014. Local multipliers and town sellers were visited throughout the selling season (April to August) and sales the previous week, the origin of each customer, the variety and quantity sold, the selling point, the type of buyer and the price were recorded, again using a questionnaire. Multipliers and town sellers were mostly illiterate and data were recorded by a team member. For town sellers, both vine purchases and sales were recorded. In 2013, sales and purchases were monitored once a week; for town sellers in 2014, this was increased to twice a week. The information on sales by town sellers comprised the current home of each customer, whether the buyer was a trader or farmer, quantity purchased, variety, price and where the vines were to be planted. The information on purchases by town sellers comprised the location of production of the supplier, whether the supplier was a multiplier or trader, the price paid, and the quantity and varieties bought. Vines were recorded as bundles of 50 vine cuttings (Fig. 3), which was the most commonly used unit both at the farm gate and at the market; when a different quantity was used, the equivalent in these bundles was recorded. The information from the multipliers seems likely to be accurate – multipliers usually sold at most once or twice a week and could remember each sale easily; town sellers often made many transactions each day and probably did not remember them all even when monitored twice a week. Sales and purchases by multipliers and sellers for each year were transferred to Excel spreadsheets to enable means, standard errors, scatter diagrams and lines giving the best fit to be calculated. Although the local multipliers and sellers interviewed were numbered only in the tens, they were monitored weekly or twice weekly over the selling period in both 2013 and 2014 and many transactions were recorded so the data involved thousands of records. Traders were very mobile, moving from local multiplier to local multiplier and could not therefore be monitored; information on them was gleaned secondhand from local multipliers and sellers.

In addition, four and six farmers growing crops during the dry season mainly for the sale of their roots but also selling their vines cheaply to traders were interviewed once in 2013 and 2014, respectively. They were otherwise unstudied because their vine sales were rare and erratic, more-or-less ‘accidental’, depending on traders or multipliers seeking their vines. These so-called ‘dry season root farmers’ usually sold vines of mature crops for a few thousand shillings to clear the land so it was easy to harvest.

The Ugandan shilling (/–) is used throughout; in 2014, $1 (USA) was equivalent to approximately 2500/–.

## Results

### Sweetpotato vine selling systems in Gulu – Actors and roles

Two main categories of vine multipliers (Table 1) were identified:

**Table 1:** Characteristics of the different types of multipliers monitored

Area (2014) of dry season sweetpotato production (ha)	Location	Number of multipliers	% Female	Average age	Year multipliers monitored	Average number of varieties grown	% hiring additional land	With phone	Buys from others	% selling to			
										Farmers	Traders	Town sellers	NGOs
a) NGO multipliers													
0.5–1.3	2 in Koro	2	50%	39 yrs	2 in 2013 2 in 2014	5	0%	100%	0	100%	0	50%	100%
b) Medium-sized local multipliers selling mostly to farmers													
0.2–0.3	2 in Bungatira	2	0%	39 yrs	1 in 2013 2 in 2014	5.5	50%	100%	0	100%	0	50%	0%
c) Small local multipliers selling mostly to farmers													
≤ 0.1	3 in Unyama 3 in Koro 5 in Bungatira	11	54%	36 yrs	5 in 2013 8 in 2014	3.1	18%	45%	0	100%	9%	0	0
d) Small local multipliers selling primarily to town sellers													
≤ 0.1	All 7 in Bungatira	7	71%	45 yrs	4 in 2013 7 in 2014	1.9	29%	0	0	71%	0	100%	0
e) Small local multipliers also buying from root producers and selling primarily to town sellers													
≤ 0.1	All 12 in Bungatira	12	92%	34	1 in 2013 12 in 2014	1.5	0	41%	100%	92%	0	100%	0

- Multipliers with irrigation growing a relatively large area (>0.5 ha) of mainly modern OFSP varieties primarily to sell the vines to NGO projects (Table 1a), referred to as ‘NGO multipliers’.
- Multipliers growing ≤0.3 ha of mainly landraces of WFSP in swamps in the dry season and selling the vines to smallscale local farmers (Table 1b-e), referred to as ‘local multipliers’.

The two NGO multipliers identified lived close together in Koro sub-county, about 6 km from Gulu on a good though unsealed road leading to Pader district (Fig. 1); they sold vines mainly to NGO and local government projects (Fig. 1). The larger one, a man, had >1 ha of dry season production and a pump for water (Table 1). They both had phones which they used for contacting buyers; they also sold to farmers, especially larger-scale farmers and rarely to the town sellers. They grew several varieties, mostly of the OFSP varieties Ejumula, Kakamega, Vita (NASPOT 9) and Kabode (NASPOT 10). They sold vines mainly in large woven plastic sacks containing 800–1000 vines. The larger NGO multiplier had formed an association with smaller multiplier neighbours and occasionally included their vines in his sales to NGOs.

The local multipliers mainly used land in two swamps to the north-east of Gulu Town (Fig. 1). Two, both men, had 0.2– 0.3 ha of dry season crops but most were women with ≤0.1 ha of dry season crops (Table 1). They mostly grew the landrace cv Ladwe Aryo, along with small amounts of cv Alero, Lalira and/or Lasoroti. None sold to NGO projects or to traders; all sold to local farmers (Table 1b-e) on farm and some sold in town to town sellers or more rarely through local markets or by the roadside (Table 1d). About half of them had a mobile phone. Some supplemented their own sales with vines bought from dry season root farmers (Table 1e). When sales were onfarm, customers (farmers or traders) would often harvest, pack and transport the vines. In contrast, for sales to town sellers or in local markets, the local multipliers had to harvest the vines, pack them in bundles and transport them to town, usually paying a motorcyclist (boda-boda) to do this. The local multipliers mainly sold individually, though some were closely related and some aggregated their production of vines to satisfy larger orders. None of the local multipliers solely multiplied vines, they also grew sweetpotato for their roots and grew other crops during the rainy season.

Both local and NGO multipliers maximized vine production of their sweetpotato in the dry season in lowlands by harvesting the vines several times. By contrast, dry season root farmers grew crops primarily to sell their roots during the off (dry)-season when these were also scarce. Some multipliers shifted to being dry season root farmers and vice-versa in different years, depending on the market, family circumstances and other factors. There appeared to be no difference in the way the crop was grown by the different multipliers; they all planted in swamps on ridges or mounds around the start of the dry season – differences were in the harvesting schedule and customers.

There were three other actors engaged in the informal selling process:

- Traders who bought vines mainly from dry season root farmers.
- Town sellers who bought vines from multipliers and traders to sell in town.
- Transporters who transported vines from local multipliers to town sellers, usually using motorbikes. The NGO multipliers or projects used transporters with trucks to take their vines to client farmers.

All the town sellers were women and they sold in an area of road ~20 m wide close to the main market in Gulu Town (Figs 1 and 2). They acted mainly as individuals but also cooperated:

- If a vine supplier for one seller brought more vines than she individually needed, she shared the supply with the others, especially if the other sellers had none to sell.
- If a customer wanted to buy more vines than any one seller could supply, they combined so that the customer gained what s/he needed.
- In paying rent jointly to the owner of the area.
- In sharing a mobile phone (but each had their own SIM cards).
- In agreeing a single mobile number for a banner used to advertise that vines were for sale because many numbers would confuse buyers.

The vines sold by the town sellers were all about 40 cm long and ready to be planted as a single cutting. These were tied neatly together in a bundle of 50 vines; 20 of these small bundles were tied together to make one large bundle (which they were willing to break up) of 1000 cuttings (Fig. 3). Town sellers all had another occupation during the off-season for selling vines; most sold fruit and vegetables then in the main market. Tying in neat bundles that customers could take away easily – an aspect of selling vegetables – seemed an innovation in the selling of vines developed specifically by Gulu town sellers. The main variety sold was Ladwe Aryo, similar to local multipliers.

Traders according to the local multipliers and town sellers were mostly smallholders with little land who bought vines from dry season root farmers. They seemed to sell vines through local markets and the town sellers and could accept the town sellers’ low purchase price (Table 3) because they had bought the vines cheaply.

No transporters were interviewed. Transporting vines was just a part of their everyday business. Multipliers told us the motorcyclists usually charged 3000/– to 5000/– for a journey and could transport 60–80 bundles at a time. Most were young men with mobile phones. Multipliers often contacted them by phone and used trusted individuals to both sell their vines to town sellers and bring back the money.

## Sales data for different multipliers

The two NGO multipliers sold large quantities of vines to NGOs (Table 2); the numbers of sales were much fewer than to farmers but their average size was >30× greater (Table 3); they rarely sold to town sellers. Their annual sales volume of vines increased about fivefold from 2013 to 2014 but their value increased only twofold, reaching an equivalent of about US$6000 for each multiplier, due to reductions in 2014 in the price they obtained for the vines (Table 3). The average price, number of bundles in each sale and value of each sale to NGOs and, to a lesser extent, to farmers fluctuated enormously (between 400/– and 1500/–per bundle) in 2013 as shown by their high standard deviations, but had settled down by 2014 to 500/– to 650/– per bundle. This is nearly twice the amount paid to local multipliers by town sellers; scatter diagrams (not shown) also showed that prices for NGOs were not reduced for either large (some single purchases were massive) or late-season purchases.

**Table 2:** Number of sales, total quantity of small bundles sold and total annual value (/−) of sales by the different multipliers. Means are given ± standard deviation when there were three or more transactions

Year	Number of multipliers	Average number of transactions/season				Average total quantity of small bundles sold to:				Average annual value (/−) of sales:			
		Farmers	NGOs	Sellers	All	Farmers	NGOs	Sellers	All	Farmers	NGOs	Sellers	All
NGO multipliers													
2013	2	11	7	0	18	660	6870	0	7530	434,300	7,249,750	0	7,684,050
2014	2	47	12	4	63	3378	25,870	75	29,323	1,547,963	13,315,555	26,500	14,890,018
Medium-sized local multipliers													
2013	1	25	0	2	27	634	0	78	712	192,000	0	35,000	227,000
2014	2	11	0	8	19	358	0	440	798	182,750	0	132,000	314,750
Small local multipliers selling only to farmers													
2013	8	16 ± 11	0	0	16 ± 11	515 ± 336	0	0	515 ± 336	150,206 ± 98,173	0	0	150,206 ± 98,173
2014	4	8 ± 4	0	0	8 ± 4	231 ± 143	0	0	231 ± 143	98,000 ± 77,365	0	0	98,000 ± 77,365
Small-scale local multipliers selling primarily to town sellers													
2013	2	4	0	14	18	104	0	466	570	40,625	0	133,950	174,575
2014	7	3 ± 3	0	15 ± 10	18 ± 10	103 ± 129	0	650 ± 513	753 ± 544	41,214 ± 51,433	0	194,929 ± 152,960	236,143 ± 163,097
Small-scale local multipliers also buying from dry season root farmers and selling primarily to town sellers													
2013	1	13	0	12	25	305	0	360	665	109,700	0	105,000	214,700
2014	12	5 ± 9	0	29 ± 18	33 ± 17	115 ± 243	0	1150 ± 809	1266 ± 772	52,125 ± 112,563	0	338,519 ± 38,533	390,644 ± 224,454

**Table 3:** The average price, number of bundles sold and the value of individual transactions by multipliers, traders and town sellers. Means are ± standard deviation

2013				For each transaction:			Total no. of observations
Sale by:	Where:	Sales to:	Sale of:	Price (/−)	No bundles	Value	
NGO multipliers	On farm	Farmers	OFSP cvs	699 ± 297	60 ± 41	39,482 ± 29,350	22
NGO multipliers	On farm	NGOs	OFSP cvs	919 ± 370	1057 ± 1247	1,115,346 ± 1,379,487	13
NGO multipliers	In town	Town sellers	Landraces	No sales were recorded this year			
Local multipliers	On farm	Smallholders	Landraces	336 ± 123	29 ± 20	8942 ± 5496	135
Local multipliers	Market/roadside	Smallholders	Landraces	420 ± 98	20 ± 14	7614 ± 3705	28
Local multipliers^*^	In town	Town sellers	Landraces	281 ± 29	32 ± 14	9339 ± 4305	55
Local multipliers	In town	Town sellers^*^	Landraces	277 ± 50	26 ± 13	7360 ± 4062	175
Local multipliers	On farm	Traders	Landraces	275 ± 96	56 ± 19	14,419 ± 4862	13
Traders^*^	In town	Town sellers^*^	Landraces	270 ± 42	27 ± 13	7475 ± 3856	113
Town sellers	In town	Customers	Landraces	372 ± 122	21 ± 9	9720 ± 24,346	312
2014							
NGO multiplier	On farm	Farmers	OFSP cvs	464 ± 94	40 ± 43	30,352 ± 25,358	93
NGO multipliers	On farm	NGOs	OFSP cvs	534 ± 91	1128 ± 883	1,024,273 ± 840,019	23
NGO multiplier	In town	Town sellers	Landraces	350 ± 100	38 ± 17	13,250 ± 6801	4
Local multipliers	On farm	Smallholders	Landraces	415 ± 99	30 ± 17	12,525 ± 8790	110
Local multipliers	Market/roadside	Smallholders	Landraces	483 ± 51	29 ± 13	13,979 ± 6119	47
Local multipliers^*^	In town	Town sellers	Landraces	295 ± 21	41 ± 20	12,238 ± 6165	448
Local multipliers	In town	Town sellers^*^	Landraces	296 ± 32	29 ± 17	8438 ± 5129	1026
Local multipliers	On farm	Traders	Landraces	No sales were recorded this year			
Traders^*^	In town	Town sellers^*^	Landraces	327 ± 90	24 ± 15	7750 ± 4736	12
Town sellers	In town	Customers	Landraces	487 ± 61	21 ± 10	10,473 ± 5379	1402

* Supplier of information

**Fig 4:**
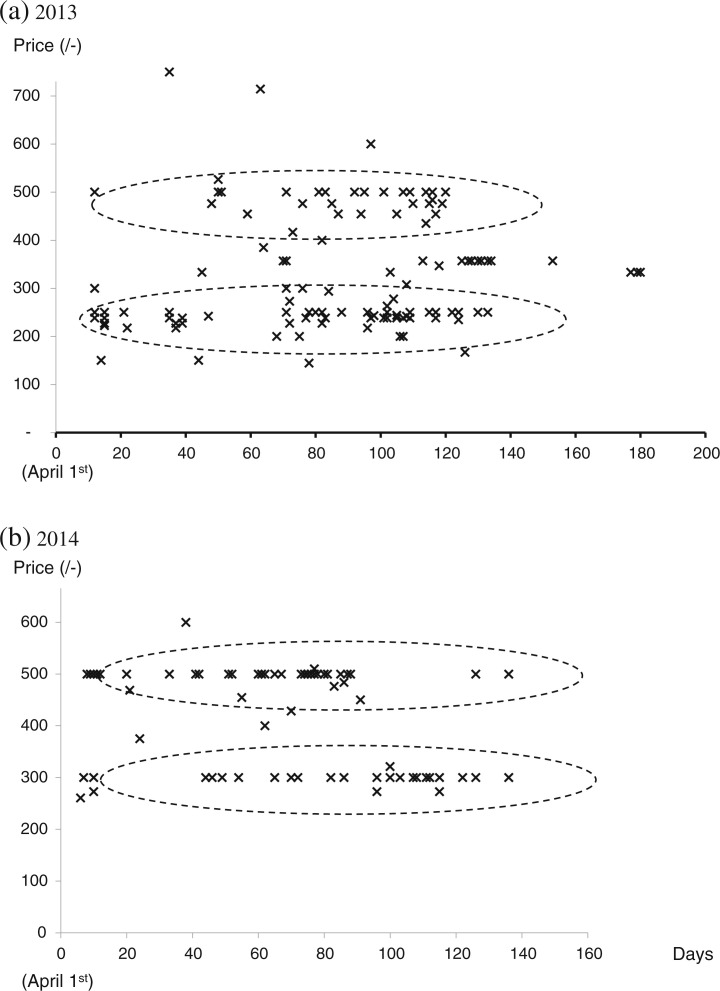
Sales of vines of landraces (Date × Price) by local multipliers on farm. The dotted ellipses enclose areas in which a large number of observations lie

All local multipliers sold on farm, some sold in local markets or to town sellers but few sold to traders (Table 2). Some also supplemented their own vine supplies by buying from dry season root farmers. The prices paid to the local multipliers varied with the type of customer and how and where they sold them. The multipliers gained the best average price, around 500/– per bundle, selling in local markets (Table 3) but to achieve this they had to harvest and transport the vines to market, wait for customers and risk failing to sell. Selling on-farm gained the next highest average price and allowed the local multiplier to do other jobs on-farm instead of waiting in the market. Interestingly, examination of scatter diagrams (Fig. 4 a and b) revealed two main prices – one at 250–300/–and another at 500/– and the multipliers told us that the price of 250–300/– per bundle involved customers harvesting the vines whereas the one priced at around 500/– per bundle involved customers pre-arranging with the multiplier (mostly by mobile phone) for the vines to be cut and packed ready to take away. This latter price was thus similar to that gained in local markets but avoided the costs of transporting the vines to market or the risk of failing to sell.

Selling the vines to town sellers meant, for the local multiplier, both accepting only 300/– per bundle and having to harvest, pack and transport them to town. Nevertheless, local multipliers sold larger quantities this way (Tables 2 & 3) and presumably adjusted their prices for this benefit. Sales on-farm declined from 2013 to 2014 whereas sales to town sellers increased such that local multipliers who did not sell to the town sellers suffered a decline in income whereas those who sold to them increased theirs. The local multipliers who also bought vines from dry season root farmers sold the most vines to the town sellers, the value of their annual sales almost doubling. The town sellers sold a bundle of vines for about 100/– more than the purchase price in 2013 but this mark-up had almost doubled by 2014. There was little variation in the number of bundles bought in individual transactions from local multipliers by different channels.

## Season of selling

**Fig 5:**
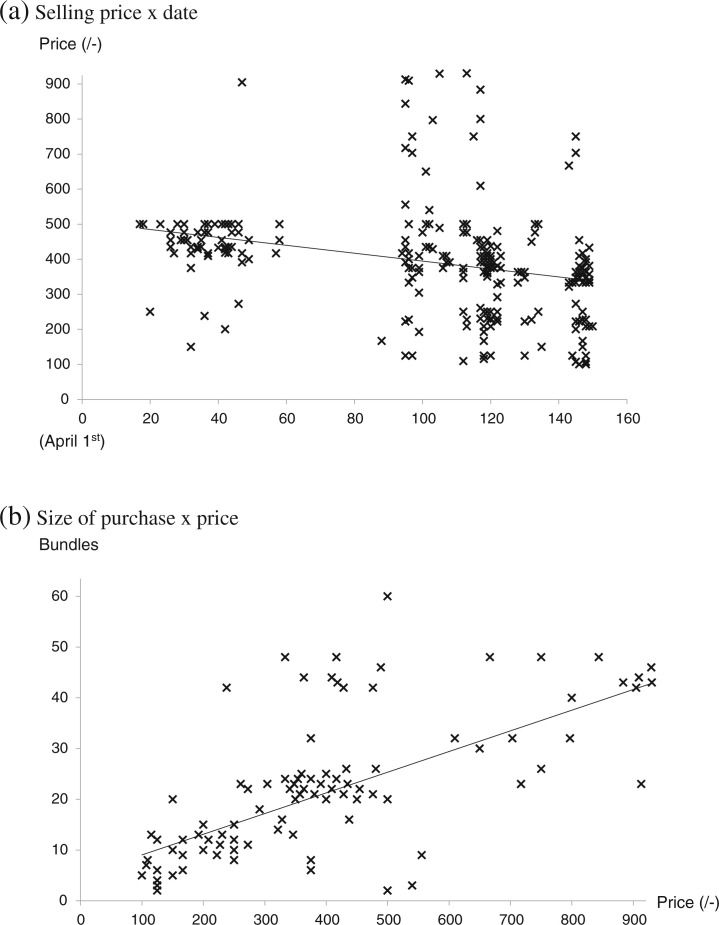
Sales of vines of landraces in 2013 by town sellers. The lines are the ‘best fit’ straight line for the data points

Sales by local multipliers started in April and were mainly in May–July; there was little difference in seasonality between sales on-farm, in markets, to traders and to town sellers (Table 4). This is about a month after the start of the rains and it seems likely that the intervening time was used to plant grain crops and for ploughing. There was little variation in seasonal prices both on-farm and in town markets, suggesting amounts available to buy were closely following the required demand (Figs. 4 and 5). In 2013, there was an extended short dry season in June, seriously disrupting planting and sales, and the prices the town sellers received from customers declined afterwards as the season progressed (Fig. 5a). No such trend was evident in 2014 (data not shown). Interestingly, smaller purchases also tended to cost less per bundle (Fig. 5b). Sales to NGOs by NGO multipliers were infrequent and tended to occur later in the season than to other customers.

**Table 4:** The percentage of sales of small bundles of vines by different multipliers in each month during the selling period

Transaction	Month (%)							Total annual sales	
	March	April	May	June	July	August	Sept	Transactions	Bundles
a) 2013									
NGO multipliers									
To farmers	0	14	50	0	32	3	2	22	1320
To NGOs	0	0	3	7	15	72	3	13	13,740
Local multipliers									
On-farm	0	11	14	24	51	1	0	135	3924
Local markets	0	0	28	18	42	12	0	28	522
To town sellers	0	0	9	29	55	7	0	55	2194
Town sellers									
Sales	0	8	16	0	51	24	0	312	6571
Purchases	0	8	16	3	42	33	0	288	7658

Transaction	Month (%)							Annual sales	
	March	April	May	June	July	August	Sept	Transactions	Bundles
b) 2014									
NGO multipliers									
To farmers	0	2	27	38	25	9	0	93	6756
To NGOs	0	25	11	36	7	21	0	23	58,471
Local multipliers									
On-farm	0	6	25	47	18	4	0	110	3244
In local markets	0	0	38	28	30	4	0	47	1376
To town sellers	1	4	32	37	18	9	0	448	19,135
Town sellers									
Sales	2	27	33	19	19	0	0	1402	29,955
Purchases	2	27	33	18	20	0	0	1026	29,459

## Vines customers

**Fig 6:**
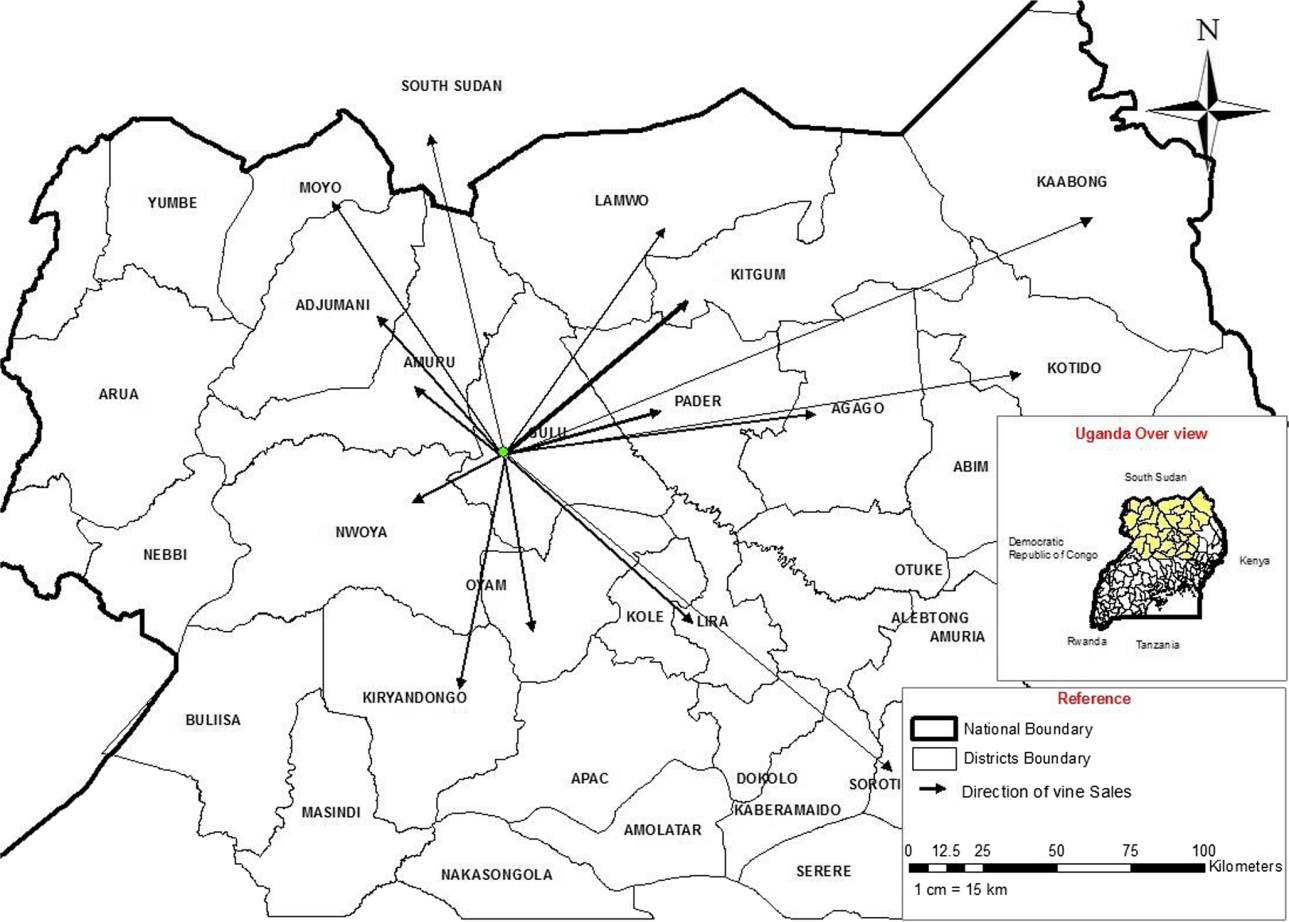
A map showing the districts other than Gulu District to which the town sellers sold vines. The relative amount sold is roughly indicated by the thickness of each arrow

The sales of vines to farmers by local multipliers were mostly to customers within a 10 km radius (Table 5). Interestingly, sales by town sellers had two peaks; one at 0–5 km away for local customers but another between 20 and 200 km away. Many of the latter were to Kitgumand Pader but they occurred in almost every direction including to South Sudan (Fig. 6). Sales to NGOs were commonly to customers located 10–100 km away.

**Table 5:** The percentages of sales (number of bundles) sold to customers living at different distances (km) from the source of the vines

Source of vines	Location of sales	Year	Distance to customer’s home (km)								Total number of:	
			0–2	3–5	6–10	11–20	21–50	51–100	101–200	>200	Bundles	Transactions
NGO multipliers	On farm (to farmers)	2013	29	45	11	2	5	9	0	0	1320	22
		2014	16	27	17	9	14	11	7	1	6756	93
	On farm (to NGOs)	2013	4	41	1	22	1	31	0	0	13,740	13
		2014	1	0	1	17	40	36	5	0	58,471	23
Local multipliers	On farm	2013	37	20	29	13	2	0	0	0	3924	135
		2014	51	28	5	1	9	1	6	0	3244	110
	Roadside + local markets	2013	36	56	8	0	0	0	0	0	522	28
		2014	16	56	2	5	21	0	0	0	1376	47
Town sellers	In town	2013	32	10	3	3	11	8	31	2	6571	312
		2014	28	11	3	1	10	6	41	1	29,955	1402

## Discussion

This description of the informal sweetpotato vine selling system in Gulu District and Town is the first detailed description of its kind in Africa. It provides details of the actors, the selling and buying prices, what is mainly bought, and how and when it is bought, and also of the magnitude and extent of the informal selling system. This description indicates that the informal selling system is providing a vital service to the local smallholders, supplying amounts of planting material which are affordable and probably convenient to them, being able both to buy in a central market and locally on-farm. It was also clear that Gulu was providing this service to many smallholders outside Gulu District, planting material from Gulu often reaching smallholders >100 km away and even in South Sudan. Data were not collected on whether price limited the quantities of vines bought and whether their amounts and quality were adequate or of the varieties required (all were landraces). Nonetheless, most purchases by farmers (but not by NGOs) were of around 30 bundles regardless of the price varying with the channel used, based perhaps largely on the amount that could be transported easily by head or on a pedal bicycle. This also was enough to plant ~0.1 ha; expanding this fourfold by using cuttings from the initial planting would generate an adequate harvest over much of the year for most households. A description of the sales of the two NGO multipliers identified in the district was necessarily more restricted but included similar data.

**Fig 7:**
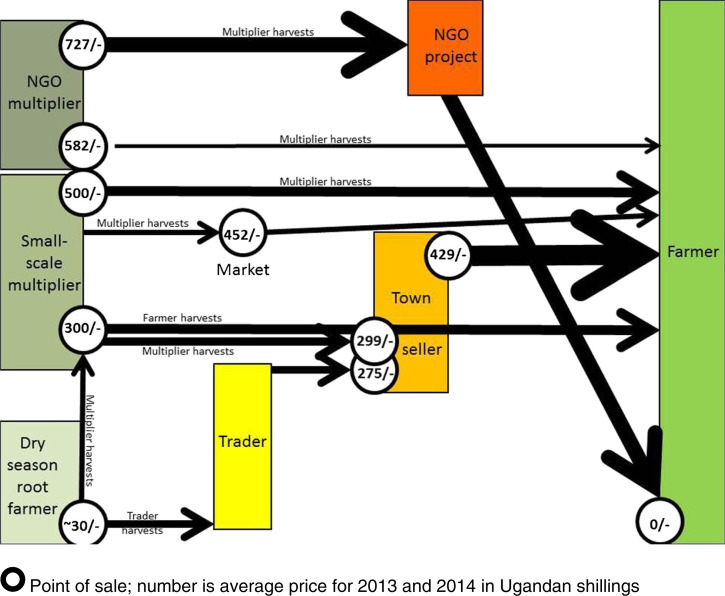
Diagram of sales channels in Gulu town and district, with approximate amounts delivered indicated by the thickness of the arrow. The numbers in the *“circles”* are the average price at that point of sale for 2013 and 2014 in Ugandan shillings

Prices along the informal seed channels in Gulu were largely consistent with costs, supply and demand (Fig. 7). The cheapest option for farmers was to cut and buy vines on a multiplier’s farm. The vines were most expensive when harvested, packed and sold by vine multipliers in local markets or similarly pre-ordered ready to take away from a multiplier’s farm. Town sellers were slightly cheaper, this may seem unexpected but the sellers bought and sold many vines, used their purchasing power to buy cheaply and to require their suppliers to cut and transport the vines to them, and also competed amongst themselves. The dry season root farmers were the cheapest source of vines of all; for them clearing the land prior to harvest was a benefit of selling the vines. Farmers couldn’t easily buy these though, as they had to be harvested at a time mainly of the dry season farmer’s choice so they were bought by traders or multiplier/aggregators to sell on. There were a few recorded accounts inconsistent with these experiences, but overall these private enterprise seed systems seemed to be examples of efficient and well-functioning marketing and trade that minimized transaction costs.

In contrast to the informal system, sales of OFSP vines by NGO multipliers often appeared to suspend normal economic behavior, suggesting a lack of information amongst at least some participants (Jagwe et al. 2010; Shepherd 1997). The NGOs bought the biggest quantities of vines, so should pay the least yet they paid the most and even massive orders were seldom at a discount. Other purchasers who bought large quantities of vines were town sellers; NGOs paid multipliers about twice the price (500–900/– per bundle) they did (300/–) and, unlike them, bought on-farm so the NGO multipliers had no transport costs. The OFSP varieties bought by NGOs shouldn’t cost more to produce than WFSP; their orange flesh seems at most only partially to explain their greater price. Aid seed systems for most crops have largely been phased out in northern Uganda (Joughin 2014) now the Lord’s Resistance Army’s activities have ceased yet the aid seed system there for OFSP vines has not and has even increased in neighbouring South Sudan, based largely on vines produced in Uganda. Half a large bag (equivalent to 10–15 small bundles) of vines is usually given to each of thousands of smallholders by NGO projects (Gibson 2013). Such large gifts disrupt private enterprise, destroying multipliers’ and others’ livelihoods. This surely goes against donors’ philosophy, suggesting that a dialogue between the NGOs, multipliers and donors is required and that NGO projects should instead use and promote the informal local multipliers, traders, and town sellers.

**Fig 8:**
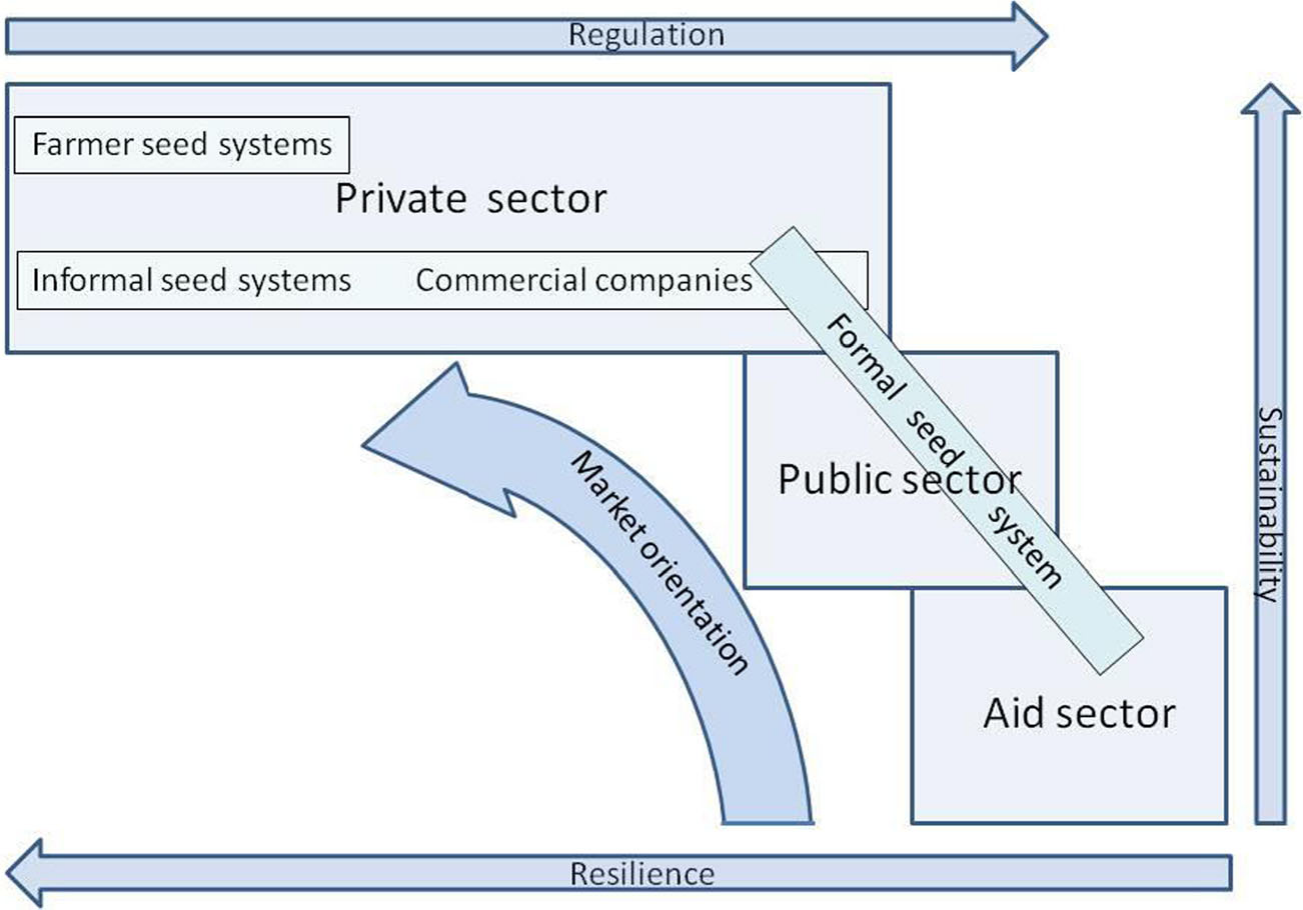
A diagram showing the relationship between resilience and sustainability in different seed systems

Seed systems can be divided into formal ones controlled by public regulations, and informal ones with no legal controls (Fig. 8), but often governed by ‘informal’ regulations such as the need to maintain a good reputation – which can be powerful. They can also be divided by ownership into the aid, public and private sectors, the private sector including both large (formal) commercial seed companies and informal systems. Seed systems are increasingly receiving attention in relation to resilience, especially around linking formal and informal sectors, with presumed overall improvements in seed systems (Louwaars and de Boef 2012). The way forward for resilience lies with informal systems (McGuire and Sperling 2013) upon which the majority of farmers rely for their seed. To achieve this challenge, different actors need to undergo some capacity development:

- Traders/sellers need to
  – increase their knowledge of the seed they sell, especially of its origin, age, certified standards etc., using competition based on advertizing the benefits these will bring to their customers as a way of driving up quality;
  – be more dynamic in accessing new varieties and competing on this too;
- Farmers need to utilize new and better ways of gaining information about seed, for example, through mobile phone usage and contacting new (for them) sources of information such as research stations and researchers.

The benefits of integrating seed systems (Louwaars and de Boef 2012) have recently gained prominence, with programs present in several African countries. An integrated approach merges the benefits of the formal sector, for example, modern varieties and the ability to disseminate certified quality seed nationally, with the strengths of informal ones that tend to contain the following features (Sperling et al. 2013):

(1) Are market-driven.
(2) Already work at scale.
(3) Rarely break down entirely.
(4) Are highly dynamic.
(5) Work everywhere and for everyone.

Aid systems generally lack all of these above features; instead they tend to work at scale only through expensively-created free handouts that are not sustained once project funding ends. A formal system is generally resilient through being sustained by taxes but it tends not to work at scale and fails to distribute planting material widely. Large-scale commercial systems are market driven, do work at scale and are highly dynamic; however, larger commercial companies require large profits and may not supply smallholders because the market is too small (FAO 2011). Like commercial systems, the informal ones are largely market driven (Table 3), work at scale (Fig. 6), and are likely to be resilient (Fig. 8) through containing large numbers of individuals working to maintain their separate livelihoods and several channels through which vines are sold (Fig. 7) (McGuire 2007; McGuire and Sperling 2013; Sperling and McGuire 2012). Long distance trading (Fig. 6) in informal systems also has demonstrated dynamism in setting up. Farmers have been reported previously to be prepared to pay for sweetpotato vines (Anonymous 2009; Gibson et al. 2009,Namanda et al. 2011). Sweetpotato is somewhat unusual both in that it is propagated vegetatively, by relatively delicate vines, rather than by seed but also that these vines are mostly destroyed during the dry season. This ensures that only the few farmers who possess access to land close to swamps or rivers can grow them then, giving them both a monopoly and an annual market, from which this cash-based system derives. We identified that local multipliers were selling about 30 small bundles (a total of about 1500 cuttings) (Table 3) to smallholders who were mostly their neighbours (Table 5) and the town sellers were mostly selling about 20 small bundles (so again the customers were mostly likely to have been smallholders), often locally but sometimes in neighbouring districts. Informal sweetpotato seed systems work for smallholders: they cannot easily supply larger farms because their scale is too small – but this is also the private sector opportunity for current NGO multipliers. Different scales of private enterprise serve different purposes, informal systems distribute plantingmaterial to smallholders and larger multipliers and commercial companies to larger farmers; only together can they claim to ‘Work everywhere and for everyone’.

The informal sector is often seen as and called a farmer seed system (reviewed: Coomes et al. 2015), with farmers producing planting material for their own use and to exchange with or sell to other farmers (Abay et al. 2011; Almekinders et al. 1994; Samberg et al. 2013). Such farmer seed systems (Aw-Hassan et al. 2008; Sperling and McGuire 2010;Witcombe et al. 2010; Takoutsing et al. 2012; Tin et al. 2011) and farmer seed businesses (de Boef and Thijssen 2010; Louwaars and de Boef 2012; Neate and Guei 2010) have often been promoted to improve the distribution of planting material. Our study of the sweetpotato seed systems in northern Uganda has not revealed a specifically farmer seed system, but instead a more diverse system comprised of specialist multipliers (some acting also as aggregators), traders, transporters and sellers (Fig. 8). As well as the town sellers detailed here, sellers have also been observed both within and at strategic junctions of trunk roads outside Arua Town (Gibson 2013). Some of these actors had not evolved from farmers; most town sellers dwelt there and had market stalls at which they sold fruit and vegetables off-season.

The sellers and local multipliers associated with them were also the most dynamic component, increasing their size of operation and connecting to farmers ≥100 km away. Despite often being portrayed as negative actors taking value away from farmers, sellers also seemed the most innovative, the neat, easily carried bundles of vine cuttings precut to planting length characteristic of Gulu not seen elsewhere in Uganda. Most aspects of the thriving informal sweetpotato seed system in Gulu also involved people acting as individuals; in contrast, cooperative seed ventures created by NGOs from farmers often failed (Gibson 2013). Early concepts of improving seed supplies supported informal systems in their entirety, often specifically mentioning traders and sellers (Bentley and Vasques 1998; Thiele 1999), yet farmer businesses (de Boef and Thijssen 2010; Louwaars and de Boef 2012; Neate and Guei 2010) eliminate these actors and perhaps along with them eliminate that vital spark, the spirit of private enterprise. Resilience, dynamism and sustainability are all the direct result of the private enterprise of individuals seeking out different market opportunities, and consumer demand for a diversity of purchasing opportunities; at least in Uganda, the public sector has failed to provide effective seed production (MAAIF 2014). Indeed, setting up seed businesses as cooperatives and insisting on farmers/smallholders being the driving force may in fact be creating exactly the cul-de-sac Almekinders and Thiele (2003) suspect exists in farmer-led seed systems. In this regard, it also seems appropriate to emphasize that the complex and cash-based trading system presented in this paper has developed in a largely subsistence crop. Whilst commercialization of a crop may be beneficial for a seed system to develop, it is clearly not essential.

## Conclusions

This work adds to evidence of how informal systems reach greater numbers of more geographically remote farmers, more sustainably through selling valued material compared with not valued material that is distributed for free through government and NGO programmes. Furthermore this significantly contributes to nutritional well-being of more remotely located families often not reached through formal systems that ‘generally fail to serve the majority of farmers’ (McGuire and Sperling 2013: 651). The informal sector can effectively improve availability and access to larger quantities and a more diverse range of varieties of planting material on a more sustainable basis to a larger number of end users. So-called ‘middlemen’ are often portrayed as negative actors taking value away from farmers. However, the presence of effective and efficient traders and sellers can contribute strongly to food security, nutrition and welfare aims by connecting often distant farmers and end users. There is, therefore, a need to focus more supportive seed sector interventions towards those systems that reach more farmers on a more sustainable basis, shifting the balance to the informal and away from the formal and NGO-based seed systems (McGuire and Sperling 2016).

There has been considerable interest over the last few decades in using informal seed systems to disseminate formally-bred varieties (Almekinders et al. 1994; Almekinders and Louwaar 2008; Thiele 1999) including sweetpotato (Gibson 2013). It has been used successfully by components of our project in Tanzania (Lukonge et al. 2015) and north-western Uganda (Obong et al. 2017). It is a cost effective way by which modern varieties (including OFSP) can be spread in developing country conditions, especially now it is known that informal systems regularly distributed seed over ≥100 km radius. Remaining challenges are that quality of planting material needs to be maintained and project funding is still needed to distribute small quantities of the new varieties to local multipliers to start the process. A protocol for Quality Declared Seed including sweetpotato has been prepared by FAO (Fajardo et al. 2010) and may provide an effective strategy for informal production of sweetpotato seed. The solution to the dissemination of new varieties could consist of a combination of rewarding national plant breeders on adoption of a variety, as in the private sector, rather than on release (Ceccarelli 2015) and having national variety trials widely dispersed on local nodal multipliers’ farms (Abay et al. 2011; Obong et al. 2017) from which the other local multipliers get access to the new varieties (Gibson 2013).

Now we understand more-or-less the current informal seed system, we continue to research factors influencing the choice of distribution channels, the use of mobile phones, and facilitating meetings between local multipliers, NGO multipliers and town sellers to explain the outcomes to date of the study and to facilitate information exchange. It is hoped that these will provide impetus for further rapid improvements and innovations to the vine supply chains.
